# The Prognostic Value of a Validated and Automated Intravascular Ultrasound-Derived Calcium Score

**DOI:** 10.1007/s12265-021-10103-1

**Published:** 2021-02-23

**Authors:** Tara Neleman, Shengnan Liu, Maria N. Tovar Forero, Eline M. J. Hartman, Jurgen M. R. Ligthart, Karen T. Witberg, Paul Cummins, Felix Zijlstra, Nicolas M. Van Mieghem, Eric Boersma, Gijs van Soest, Joost Daemen

**Affiliations:** Department of Cardiology, Thoraxcenter, Erasmus University Medical Center, Rotterdam, The Netherlands

**Keywords:** Coronary artery disease, Coronary calcification, Intravascular ultrasound, Automated quantification, Prognosis

## Abstract

**Background:**

Coronary calcification has been linked to cardiovascular events. We developed and validated an algorithm to automatically quantify coronary calcifications on intravascular ultrasound (IVUS). We aimed to assess the prognostic value of an IVUS-calcium score (ICS) on patient-oriented composite endpoint (POCE).

**Methods:**

We included patients that underwent coronary angiography plus pre-procedural IVUS imaging. The ICS was calculated per patient. The primary endpoint was a composite of all-cause mortality, stroke, myocardial infarction, and revascularization (POCE).

**Results:**

In a cohort of 408 patients, median ICS was 85. Both an ICS ≥ 85 and a 100 unit increase in ICS increased the risk of POCE at 6-year follow-up (adjusted hazard ratio (aHR) 1.51, 95%CI 1.05–2.17, *p* value = 0.026, and aHR 1.21, 95%CI 1.04–1.41, *p* value = 0.014, respectively).

**Conclusions:**

The ICS, calculated by a validated automated algorithm derived from routine IVUS pullbacks, was strongly associated with the long-term risk of POCE.

**Graphical abstract:**

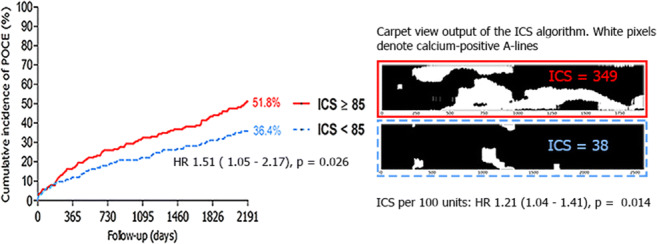

**Supplementary Information:**

The online version contains supplementary material available at 10.1007/s12265-021-10103-1.

## Introduction

Calcification is the end result of apoptosis of smooth muscle cells and macrophages and proved to be associated with advanced stages of atherosclerosis [1]. Vascular calcification is an established independent predictor of all-cause mortality and cardiovascular events in a general population [2].

The coronary artery calcium score as detected by computed tomography emerged as the strongest predictor of incident coronary artery disease in an asymptomatic population [3–7]. Besides its predictive value, the presence and extent of coronary calcium are also of importance in light of percutaneous coronary intervention (PCI). Presence of coronary calcifications is considered to be a major predictor of suboptimal stent expansion, which in turn increases the risk for target vessel failure [8–10].

Coronary angiography lacks sensitivity and precludes quantification of the extent of coronary calcium. Intravascular imaging techniques like intravascular ultrasound (IVUS) and optical coherence tomography (OCT) have a higher sensitivity to detect coronary calcification but lack the ability to automatically quantify calcium burden [11, 12]. While several algorithms have been assessed to automatically detect and quantify the extent of coronary calcification, there is a lack of data linking validated IVUS-derived calcium scores to clinical outcome [13–15].

We recently developed, trained, and validated a novel algorithm to automatically detect and quantify coronary artery calcium on IVUS using machine learning techniques [16]. The objective of the present study is to assess the clinical applicability of this algorithm to determine patient outcomes in a large local PCI and IVUS registry.

## Methods

### Inclusion and Exclusion

In this retrospective single-center cohort study, we enrolled patients who underwent coronary angiography with pre-procedural IVUS imaging. We screened patients for eligibility between January 2008 and January 2018. Patients were included if motorized IVUS pullbacks over a length ≥ 40 mm in a native coronary artery were available. IVUS-related exclusion criteria were (1) the presence of stent struts, (2) poor imaging quality, and (3) catheter in false lumen. Only one pullback per patient was included; in case of availability of pullbacks from different coronary arteries, analyses were restricted to the longest analyzable pullback. Also, in case of multiple pullbacks from the same artery, analyses were restricted to the longest pullback.

### Index Procedure

Invasive studies evolved per standard practice and IVUS imaging was used according to operator’s preference. IVUS imaging was performed with a 40 MHz IVUS catheter (Atlantis SR Pro2 or OptiCross, Boston Scientific Corporation, Natick, Mass) at a pullback speed of 0.5 mm/s. IVUS pullbacks were stored as dicoms in a dedicated local database and were analyzed offline.

### IVUS-Calcium Score

We recently developed and validated an automatic calcium detection algorithm to quantify coronary calcium on IVUS pullbacks [16]. Coronary calcium as visualized by IVUS was defined as a sharp white border accompanied by a dark acoustic shadowing [17]. A support vector machine was trained and tested on 35 pullbacks with manually annotated calcium arcs to detect this feature of calcium automatically per A-line in each frame. Reported accuracy, precision, and recall were 0.89, 0.92, and 0.85, respectively [16]. The IVUS-calcium score (ICS) was computed per pullback and defined as the number of calcium-positive A-lines divided by the total numbers of A-lines times 1000.

### Data Collection Procedure

The primary endpoint was Academic Research Consortium-2 defined Patient-Oriented Composite Endpoint (POCE): a composite endpoint consisting of all-cause mortality, any stroke, any myocardial infarction, and any revascularization [18]. Secondary endpoints included target vessel revascularization, target vessel myocardial infarction, and individual components of POCE. Target vessel was defined as the vessel that was imaged with IVUS during the index procedure and that was used for computation of the ICS: the study vessel. Survival data were obtained through municipal civil registry checks. Data on outcome measures and baseline characteristics were obtained from local electronic patient records whenever available. A health questionnaire was sent to all living patients evaluating re-admission and cardiovascular and cerebrovascular events. Patient-reported outcomes were verified with source documentation from referring hospitals. General practitioners, referring cardiologists, and patients were contacted as necessary for additional information. Ethical approval for this study was waived by the Institutional Review Board of the Erasmus Medical Center because of the retrospective nature of the clinically collected data.

### Data Analysis

Normality of continuous variables was tested using the Shapiro-Wilk test. Normally distributed variables are displayed as mean ± standard deviation (SD), whereas non-normally distributed variables are displayed as median and interquartile range (IQR; 25th–75th percentile). Categorical variables are displayed as counts and percentages. In this first clinical validation study, we decided to evaluate the ICS both as a categorical and a continuous variable. The ICS was dichotomized according to the median of 85. Differences in baseline continuous variables between patients with an ICS < 85 and an ICS ≥ 85 were compared with the independent Student’s *t* test or the Mann-Whitney *U* test, and differences in categorical covariates between patients with an ICS < 85 and an ICS ≥ 85 were evaluated with the Pearson’s chi-square test or Fischer’s exact test, as appropriate. We performed uni- and multivariate linear regression to identify factors associated with ICS. Because residuals of regression analyses using the ICS directly were non-normally distributed, we applied a square root transformation to the ICS (√ICS) for this analysis (standard log transformation also did not meet linear regression assumptions). Baseline patient characteristics with *p* value < 0.1 in univariate linear regression for √ICS were included in the full multivariate model.Patients lost to follow-up were considered being at risk for the event until 6 years (2191 days) after the index procedure or until the day of the last contact, at which point they were censored. In case of multiple events, the first event was taken into account. The Kaplan-Meier method was used to estimate survival functions for patients with ICS < 85 and ICS ≥ 85. Differences in survival time distributions between these groups of patients were assessed using the log-rank test. Uni- and multivariate Cox regression models were built to study the relationship between ICS and the study endpoints. Multivariate adjustment was carried out to remove potential bias introduced by confounding variables and other influential factors. Selection of variables for multivariate adjustment in each model was performed according to clinical relevance in literature and presence of a univariate association in our own results. All covariates were checked for satisfying the proportional hazard assumption by Schoenfeld residual tests. Competing risks were taken into account by using the cumulative incidence function to calculate cumulative incidences and cause-specific hazard regression to obtain hazard ratios. A two-sided *p* value of < 0.05 was considered as statistically significant. Statistical analyses were performed using IBM SPSS Statistics for Windows, version 25.0 (IBM Corp., Armonk, NY, USA) and R (R Core Team 2019; version 3.5.2, packages: ggplot2, survival, splines, cmprsk).

## Results

### Screening and Inclusion

Between January 2008 and January 2018, 1265 IVUS pullbacks were identified from 892 patients undergoing coronary angiography plus pre-procedural IVUS imaging. In 347 pullbacks, stent struts from previously implanted stents were present, and 355 pullbacks were too short (< 40 mm). In total, 408 patients were included in the analysis. Detailed information on the number of exclusions can be found in the Electronic Supplementary Material (Supplementary Table 1).

### Baseline Characteristics

**Fig. 1:**
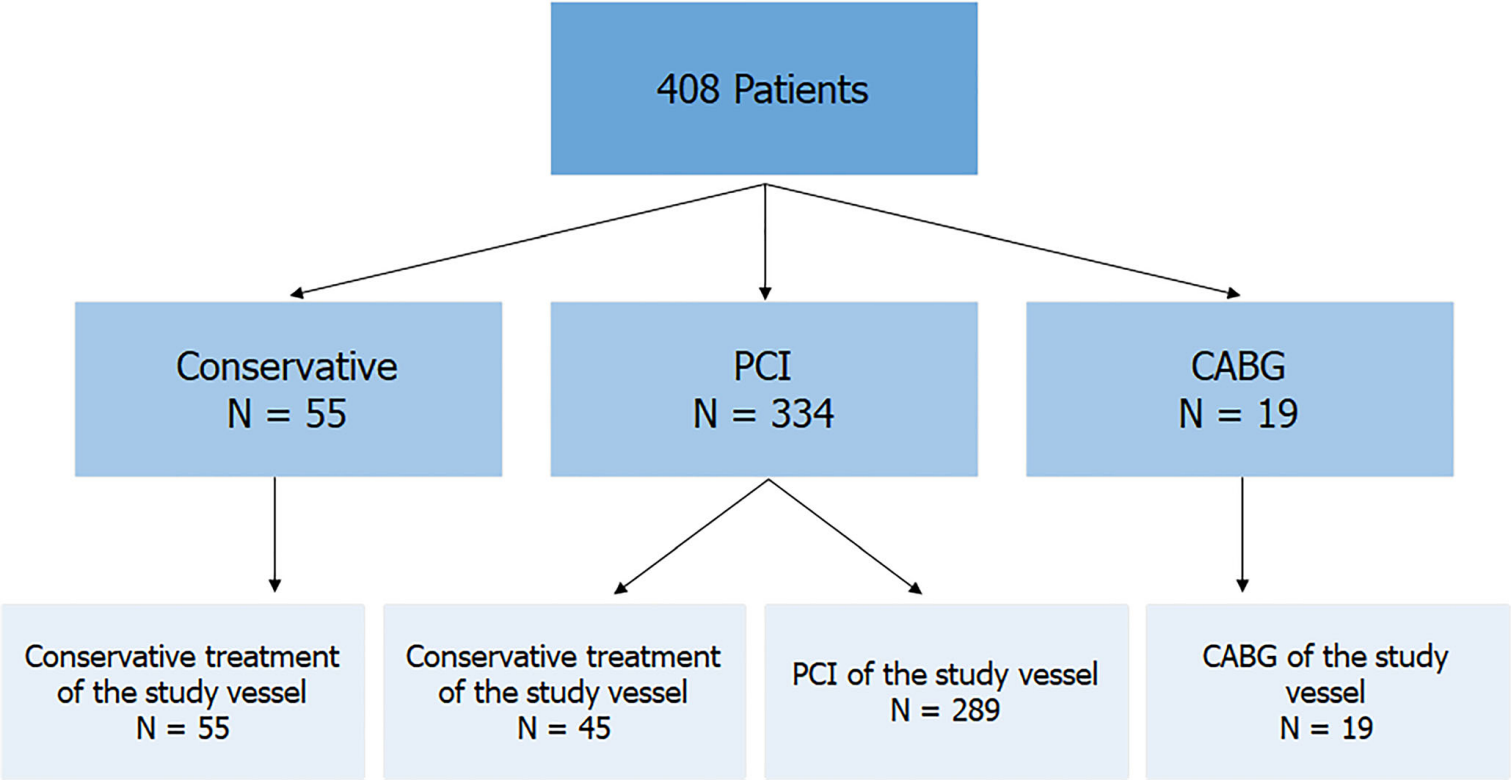
Flowchart indicating type of treatment following enrolment. Abbreviations: CABG = coronary artery bypass graft, IVUS = intravascular ultrasound, PCI = percutaneous coronary intervention

The median age of the cohort was 65.7 years (IQR 57.2–72.2) and 72.5% were male. Stable angina was the presenting symptom in 56.4% and 86.5% of patients underwent subsequent revascularization (PCI (*n*=334; 81.9%) or coronary artery bypass grafting (CABG) (*n*=19; 4.7%)) (Fig. 1). Renal impairment (eGFR < 60 ml/min) was present in 18.9% of patients.

**Fig. 2:**
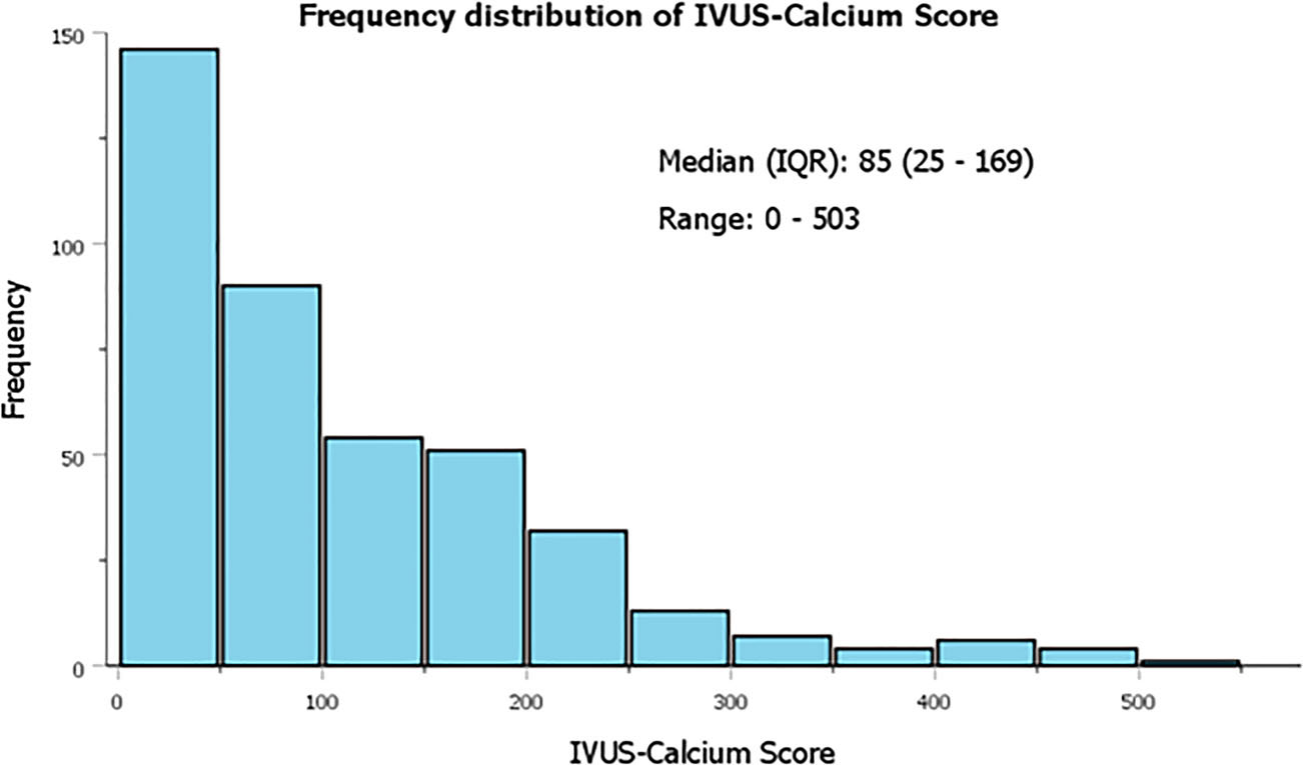
The frequency distribution of ICS. Median ICS was 85 (IQR 25–169) and ranged from 0 to 503. Abbreviations: ICS = IVUS-calcium score, IQR = interquartile range, IVUS = intravascular ultrasound

The median ICS was 85 (IQR 25–169) and ranged from 0 to 503 (Fig. 2). Patients with an ICS ≥ 85 were older, more frequently suffered from hypertension and hypercholesterolemia, more often had a previous stroke, peripheral artery disease, and renal impairment, and were more often referred for CABG. Patients with an ICS < 85 more often presented with acute coronary syndromes (Table 1).

**Table 1:** Baseline characteristics of total cohort, patients with an ICS < 85 and ICS ≥ 85. Values are displayed as median (IQR) or *n* (%)

	All patients *N* = 408	ICS < 85 *N* = 204	ICS ≥ 85 *N* = 204	*P* value
Patient level				
Age in years	65.7 (57.2–72.2)	62.7 (53.9–70.2)	68.0 (61.1–74.2)	< 0.001
Male gender	296 (72.5%)	147 (72.1%)	149 (73.0%)	0.82
Hypertension	260 (63.7%)	115 (56.4%)	145 (71.1%)	0.002
Hypercholesterolemia	226 (55.4%)	96 (47.1%)	130 (63.7%)	0.001
Statin treatment	233 (57.1%)	106 (54.4%)	127 (66.1%)	0.018
Diabetes mellitus	84 (20.6%)	34 (16.7%)	50 (24.5%)	0.050
Positive familial history	152 (37.3%)	80 (39.2%)	72 (35.3%)	0.41
Smoking	165 (40.4%)	86 (42.2%)	79 (38.2%)	0.48
Previous MI	104 (25.5%)	52 (25.5%)	52 (25.5%)	1
Previous PCI	106 (26.1%)	53 (26.0%)	53 (26.0%)	1
Previous CABG	16 (3.9%)	7 (3.4%)	9 (4.4%)	0.61
Previous stroke	22 (5.4%)	4 (2.0%)	18 (8.8%)	0.003
Previous PAD	42 (10.3%)	13 (6.4%)	29 (14.2%)	0.006
Renal impairment (eGFR < 60 ml/min)	77 (18.9%)	26 (13.7%)	51 (25.8%)	0.003
eGFR (ml/min)	79 (63–90)	83 (68–93)	74 (58–88)	< 0.001
Presentation with ACS	178 (43.6%)	103 (50.5%)	75 (36.8%)	0.005
Index treatment				0.005
PCI performed at baseline	334 (81.9%)	169 (82.8%)	165 (80.9%)	
CABG performed at baseline	19 (4.7%)	3 (1.5%)	16 (7.8%)	
Conservative treatment at baseline	55 (13.5%)	32 (15.7%)	23 (11.3%)	
Vessel level				
Study vessel				0.13
Left anterior descending	262 (64.2%)	123 (60.3%)	139 (68.1%)	
Left circumflex	59 (14.5%)	33 (16.2%)	26 (12.7%)	
Right coronary artery	77 (18.9%)	45 (22.1%)	32 (15.7%)	
Left main stem	10 (2.5%)	3 (1.5%)	7 (3.4%)	
Study vessel revascularized at index procedure (either PCI or CABG)	308 (75.5%)	144 (71.1%)	164 (80.9%)	0.021

*ACS* acute coronary syndrome, *BMS* bare metal stent, *BRS* bioresorbable scaffold, *CABG* coronary artery bypass graft, *DES* drug-eluting stent, *eGFR* estimated glomerular filtration rate, *ICS* IVUS-calcium score, *IQR* interquartile range, *IVUS* intravascular ultrasound, *MI* myocardial infarction, *PAD* peripheral artery disease, *PCI* percutaneous coronary intervention

A total of 308 (75.5%) vessels were subsequently treated with either PCI (*n*=289; 70.1%) or CABG (*n*=19; 4.7%) (Fig. 1). The remaining 100 vessels (24.5%) were a mix of non-culprit coronary arteries (*n*=45) and vessels from patients that were finally treated conservatively (*n*=55). Study vessels with an ICS ≥ 85 were more often revascularized compared with vessels with an ICS < 85 (Table 1). The distribution of study vessels was similar between the groups.

A total of 57.1% (233/408) patients were on statin therapy at the time of the index procedure. Patients on statin treatment had significantly higher ICS as compared with statin naive patients (121 ± 101.4 vs 91 ± 103.2, *p* = 0.006) and patients with ICS ≥ 85 appeared to be more often on statin therapy as compared with patients with ICS < 85 (66.1% vs 54.4%, *p* = 0.018).

### Multivariate Linear Regression

Median √ICS was 9.2 (IQR 5.1–15.7) and ranged from 0 to 22.4. Increasing age and previous stroke remained as significant explanatory variables for √ICS in the multivariate model. Previous stroke was associated with a mean difference of 2.46 units of √ICS (*p* = 0.029), whereas each year of increase in age was associated with a mean increase of 0.07 units of √ICS (*p* = 0.002) (Table 2). See Supplementary Figure 1 (Electronic Supplementary Material) for a visual interpretation of the significant covariates when back-transformed to the linear scale.

**Table 2:** Uni- and multivariate linear regression models for √ICS

Linear regression √ICS	Univariate			Multivariate		
	*β*	95% CI *β*	*P* value	*β*	95% CI *β*	*P* value
Gender	0.22	−0.90–1.34	0.70			
Age, in years	0.11	0.07–0.15	< 0.001	0.07	0.03–0.12	0.002
Hypertension	1.65	0.63–2.68	0.002	0.89	−0.18–1.96	0.10
Hypercholesterolemia	1.53	0.54–2.53	0.002	0.92	−0.11–1.94	0.079
Diabetes mellitus	1.52	0.30–2.75	0.015	1.02	−0.19–2.22	0.097
Positive familial history	−0.62	−1.65–0.41	0.24			
Smoking	−0.05	−1.07–0.97	0.92			
Previous MI	0.04	−1.10–1.19	0.94			
Previous PCI	0.23	−0.91–1.36	0.70			
Previous CABG	1.15	−1.42–3.72	0.38			
Previous stroke	3.87	1.69–6.05	0.001	2.46	0.25–4.68	0.029
Previous PAD	2.37	0.74–4.00	0.004	1.10	−0.54–2.74	0.19
eGFR	−0.04	−0.06 to −0.02	0.001	−0.02	−0.04–0.01	0.25
Presentation with ACS	−1.35	−2.35 to −0.35	0.008	−0.87	−1.88–0.14	0.091
Study vessel LAD	0.43	−0.61–1.47	0.42			

*ACS* acute coronary syndrome, *CABG* coronary artery bypass graft, *CI* confidence interval, *eGFR* estimated glomerular filtration rate, *ICS* IVUS-calcium score, *LAD* left anterior descending, *MI* myocardial infarction, *PAD* peripheral artery disease, *PCI* percutaneous coronary intervention

### Survival Analysis

**Fig. 3:**
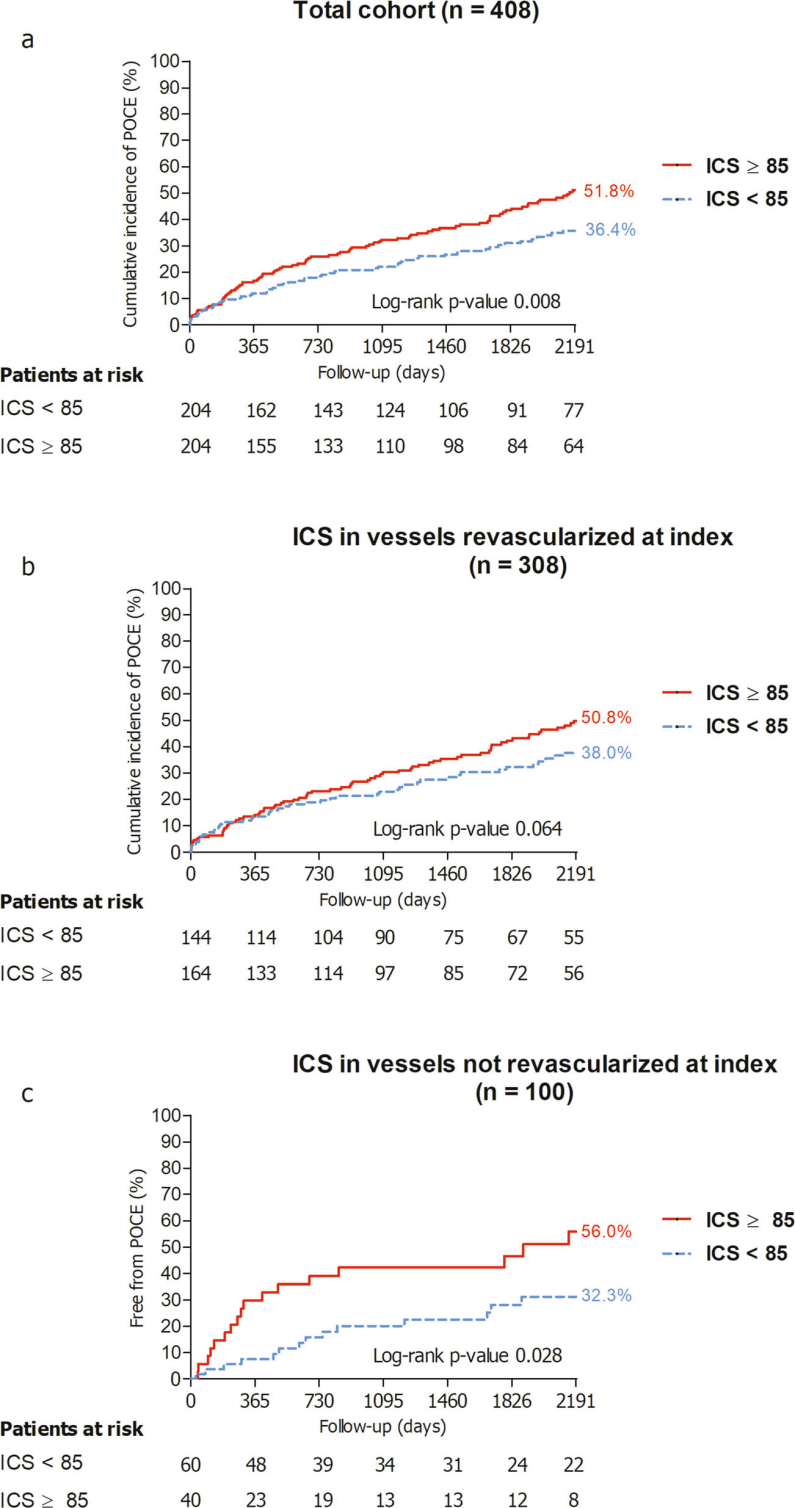
Cumulative incidence curves for POCE for the total cohort (**a**), the subgroup of patients in whom the study vessel was revascularized (**b**), and the subgroup of patients in whom the study vessel was not revascularized (**c**). The red solid lines represent the cumulative incidence curves for patients with an ICS ≥ 85 and the dashed blue lines represent the cumulative incidence curves for patients with an ICS < 85. The cumulative incidence curves of patients with an ICS ≥ 85 versus ICS < 85 were compared using the log-rank test. Abbreviations: ICS = IVUS-calcium score, POCE = patient-oriented composite endpoint

The median follow-up was 2166 (987–3299) days. During a follow-up of 6 years (2191 days), POCE occurred in 152 patients (cumulative incidence 44.4%). The cumulative incidence of POCE for patients with ICS ≥ 85 was 51.8% versus 36.4% for patients with ICS < 85 (*p* = 0.008) (Fig. 3).

Both an ICS ≥ 85 and each 100 units increase in the ICS were associated with a significant increase in the risk of POCE: the adjusted hazard ratio (aHR) for ICS ≥ 85 was 1.51 (95% CI 1.05–2.17, *p* value = 0.026) and for each 100 units increase in ICS 1.21 (95% CI 1.04–1.41, *p* value = 0.014) (Table 3). A 100 unit increase in the ICS appeared to be associated with a significantly increased rate of target vessel revascularization: the aHR for each 100 units increase in ICS was 1.37 (95% CI 1.06–1.77, *p* value = 0.017).Detailed multivariate models for the prognostic value of the ICS on the individual components of POCE can be found in Supplementary Table 2 (Electronic Supplementary Material).

**Table 3:** Event rates and multivariate Cox regression models for the composite endpoint POCE and the individual endpoints of POCE for both ICS ≥ 85 and ICS as a continuous covariate (ICS_100 units_). Values are *n* (cumulative incidence) unless specified otherwise. The multivariate model for POCE was stratified on diabetes due to non-proportional hazards. Detailed information and tables of full models can be found in Supplementary Table 2 (Electronic Supplementary Material)

	Number of patients with event at 6-year follow-up			ICS ≥ 85		ICS_100 units_	
Event of interest	Total (*n*=408)	ICS < 85 (*n*=204)	ICS ≥ 85 (*n*=204)	HR (95% CI)	*p* value	HR (95% CI)	*p* value
POCE	152 (44.4%)	61 (36.4%)	91 (51.8%)	1.51 (1.05–2.17)	0.026	1.21 (1.04–1.41)	0.014
Any revascularization	80 (23.2%)	37 (22.1%)	43 (24.2%)	1.31 (0.82–2.10)	0.26	1.23 (1.00–1.51)	0.055
PCI	71 (20.8%)	34 (20.4%)	37 (21.1%)	1.25 (0.77–2.05)	0.37	1.20 (0.96–1.49)	0.11
CABG	13 (3.6%)	5 (2.9%)	8 (4.3%)	1.61^a^ (0.53–4.91)	0.41	1.30^a^ (0.82–2.06)	0.27
Target vessel revascularization	42 (12.0%)	18 (10.4%)	24 (13.4%)	1.68 (0.88–3.19)	0.11	1.37 (1.06–1.77)	0.017
Any myocardial infarction	31 (9.3%)	12 (7.3%)	19 (11.0%)	1.44 (0.69–3.02)	0.33	1.28 (0.95–1.73)	0.10
Target vessel myocardial infarction	20 (5.7%)	8 (4.5%)	12 (6.7%)	1.47 (0.60–3.60)	0.40	1.29 (0.90–1.85)	0.17
Any stroke	14 (4.1%)	5 (2.9%)	9 (5.3%)	1.80^a^ (0.60–5.38)	0.29	1.37^a^ (0.89–2.10)	0.15
All-cause mortality	68 (18.9%)	21 (12.0%)	47 (25.6%)	1.55 (0.91–2.64)	0.10	1.16 (0.94–1.43)	0.17

*CABG* coronary artery bypass graft, *CI* confidence interval, *HR* hazard ratio, *ICS* IVUS-calcium score, *PCI* percutaneous coronary intervention, *POCE* patient-oriented composite endpoint

^a^Due to a limited number of events, only univariate cox regression could be provided

In the study vessel revascularized at index cohort (n = 308) and study vessel not revascularized at index cohort (*n* = 100), the cumulative incidences of POCE for patients with ICS ≥ 85 versus patients with ICS < 85 were 50.8% versus 38.0% (*p* = 0.064) and 56.0% versus 32.3% (*p* = 0.028) (Fig. 3).

## Discussion

In the present study, we assessed the prognostic value of a validated and automated IVUS-derived calcium score, the ICS, on POCE at 6 years. We demonstrated that (1) patients with an ICS ≥ 85 had an overall higher cardiovascular risk profile and (2) the ICS both as a dichotomized (ICS ≥ 85) and as a continuous score significantly predicts POCE.

Apoptosis of smooth muscle cells and macrophages are believed to induce the formation of microcalcifications (0.5–15 μm) in the intimal layer of the coronary artery [19, 20]. Fusion of these microcalcifications and further progression of calcification over time results in calcified plaques formed by calcified sheets or plates that can be identified with computed tomography and during invasive coronary angiography. Mildly calcified plaques are common and moderate to severe calcifications can be found in up to 18% of patients with an inherent risk for increased event rates [21].

Our findings support the evidence that patients with a greater extent of coronary calcification have a higher cardiovascular risk profile [6, 21–24]. We found that patients with an ICS ≥ 85 were more likely to be older, have hypercholesterolemia, hypertension, renal impairment, a previous stroke, previous peripheral arterial disease, present with stable angina (as compared with acute coronary syndrome), and undergo subsequent coronary revascularization. After multivariate adjustment, age and previous cerebrovascular accident remained independent predictors for ICS, which is presumably due to our relatively small sample size. Several studies have demonstrated that the coronary artery calcium score is a strong predictor for cardiovascular events in asymptomatic individuals [2–4, 6, 25, 26]. To the best of our knowledge, the ICS is the first validated score for coronary artery calcification linked to cardiovascular events since the introduction of the computed tomography-derived coronary artery calcium score 30 years ago [5]. In fact, we found a 51% increased risk of POCE in patients with an ICS above the median of 85. The fact that we have found that a calcium score based on one coronary artery alone is associated with impaired patient outcome reflects the systematic nature of atherosclerotic cardiovascular disease. Caution is warranted when comparing the computer tomography-derived coronary artery calcium score and the ICS. The non-invasively obtained coronary artery calcium score has its particular value in decision-making in cardiovascular risk assessment, in particular in the asymptomatic population, while the ICS has been derived from invasive intracoronary evaluation in patients with suspected coronary artery disease [27].

An apparent heterogeneity was found in the predictive value of an ICS (cut-off 85) for POCE in vessels that were target of revascularization versus non-culprit vessels (Fig. 3). The latter could be explained by a higher ICS in vessels that were target to revascularization as compared with non-culprit vessels (median ICS 91 versus median ICS 64, respectively). Moreover, higher ICS (each 100 units increase) proved to be an independent predictor of target vessel revascularization. Nevertheless, these findings must be considered as explorative and hypothesis-generating as our sample size was too small to (adequately) control for confounding variables in these analyses.

Intravascular imaging using either IVUS or OCT has proven to be superior to coronary angiography and multi-slice computed tomography for the detection of coronary calcification [11, 12, 28]. Non-automated assessment of coronary calcium for both clinical and research purposes is time-consuming. Recently, several algorithms have been proposed to automatically detect plaque features in intravascular imaging techniques. The concept of virtual histology (VH)-IVUS was first introduced in 2002 and demonstrated to be able to detect plaque characteristics in non-culprit coronary arteries that predict major adverse cardiovascular events [29–31]. At present, the use of VH-IVUS has practically been abandoned due to repetitive questions on the validity of the algorithm and lack of ability of VH-IVUS to alter patient management.Previous automated classification tools for detection of calcium in 20 MHz IVUS pullbacks and OCT have been proposed as well, but have mainly focused on tissue type segmentation in cross-sectional images [13, 14, 32]. This study is the first to derive a clinically relevant score and to validate this score against clinical outcome.

On a patient level, the automatic detection of coronary calcification might improve our ability to classify the risk of an individual patient. Next to presentation with ACS, a history of CABG, and eGFR, ICS proved to be among the strongest predictors of POCE at 6 years. On a lesion level, the ICS might play an important role in future algorithms for lesion preparation in an era where the treatment armamentarium for calcified coronary artery disease is rapidly expanding. The direct relation between lesion-specific ICS and stent expansion might shed further light on this issue and is therefore subject of further study. Another potential application of the ICS includes serial plaque imaging assessing the effect of pharmacological interventions on calcified plaque volume.

At present, the use of the ICS is restricted to patients referred for coronary angiography in which IVUS catheters from vendors in which the algorithm has been validated are used. Moreover, the use of the ICS is restricted to native coronary artery disease and vessels that have not been subject to prior stenting. Dedicated future studies towards the feasibility of implementation of our algorithm in commercially available IVUS systems are needed which limits current clinical use. Nevertheless, as mentioned above, several clinical scenarios could be envisaged in which the ICS could be of use in both clinical practice as well as research settings.

### Limitations

Strengths of our study include the access to a large local IVUS and PCI database and a validated algorithm built to detect specific A-line features and automatically generate a calcium score. Given the lack of a need for manual contour detection, the algorithm provides no data on atheroma volume precluding any statements on the prognostic utility of ICS over plaque burden. Moreover, given the inability of IVUS to assess calcium thickness, the ICS is not based on total calcium volume in the coronary artery, but on calcium arc and length only.

Secondly, strict inclusion criteria and retrospective screening led to a limited sample size, which potentially impacted the scope of the present analysis. Thirdly, we decided to dichotomize the ICS according to its median value in this first clinical validation study, similar to previous published work on the lipid core burden index [33]. This does not imply that an ICS ≥ 85 is the best cut-off value. Finally, while ICS was significantly higher in patients taking statins, we could not ascertain a definite causal relation and exclude the fact that the relation found was a reflection of the higher overall risk profile in patients with a higher ICS. Yet, despite these limitations, we could demonstrate that the ICS significantly increases the risk of cardio-cerebrovascular events.

## Conclusion

The ICS is a novel scoring tool that automatically quantifies the extent of coronary calcification on IVUS and is significantly related to impaired cardiovascular and cerebrovascular outcomes in an all-comer population of patients undergoing coronary angiography and IVUS imaging.

## Supplementary information

ESM 1 Visual representation of relationship between ICS, age and a previous stroke on the linear scale. Regression coefficients were originally estimated for √ICS because of violation of the normality and homoscedasticity assumptions when using ICS, and then back-transformed by squaring the regression formula. Pink diamond shaped points represent the ICS and age of patients without a previous stroke. Blue triangle shaped points represent the ICS and age of patients with previous stroke. The blue and the pink line represent the regression line for the ICS of patients with varying age with or without a previous stroke, respectively. Abbreviations: ICS = IVUS-Calcium score. (PNG 3114 kb)

High resolution image (TIF 12461 kb)

ESM 2 (DOCX 19 kb)

## Abbreviations

aHR: Adjusted hazard ratio
BMS: Bare metal stent
BRS: Bioresorbable scaffold
CABG: Coronary artery bypass grafting
CI: Confidence interval
ICS: IVUS-calcium score
IQR: Interquartile range
IVUS: Intravascular ultrasound
OCT: Optical coherence tomography
PCI: Percutaneous coronary intervention
POCE: Patient-oriented composite endpoint
SD: Standard deviation
