# Supplementary figures and images for: The Prognostic Value of a Validated and Automated Intravascular Ultrasound-Derived Calcium Score
Source: J Cardiovasc Transl Res. 2021 Feb 23;14(5):992–1000. doi: 10.1007/s12265-021-10103-1 (PMC8575752; doi:10.1007/s12265-021-10103-1)

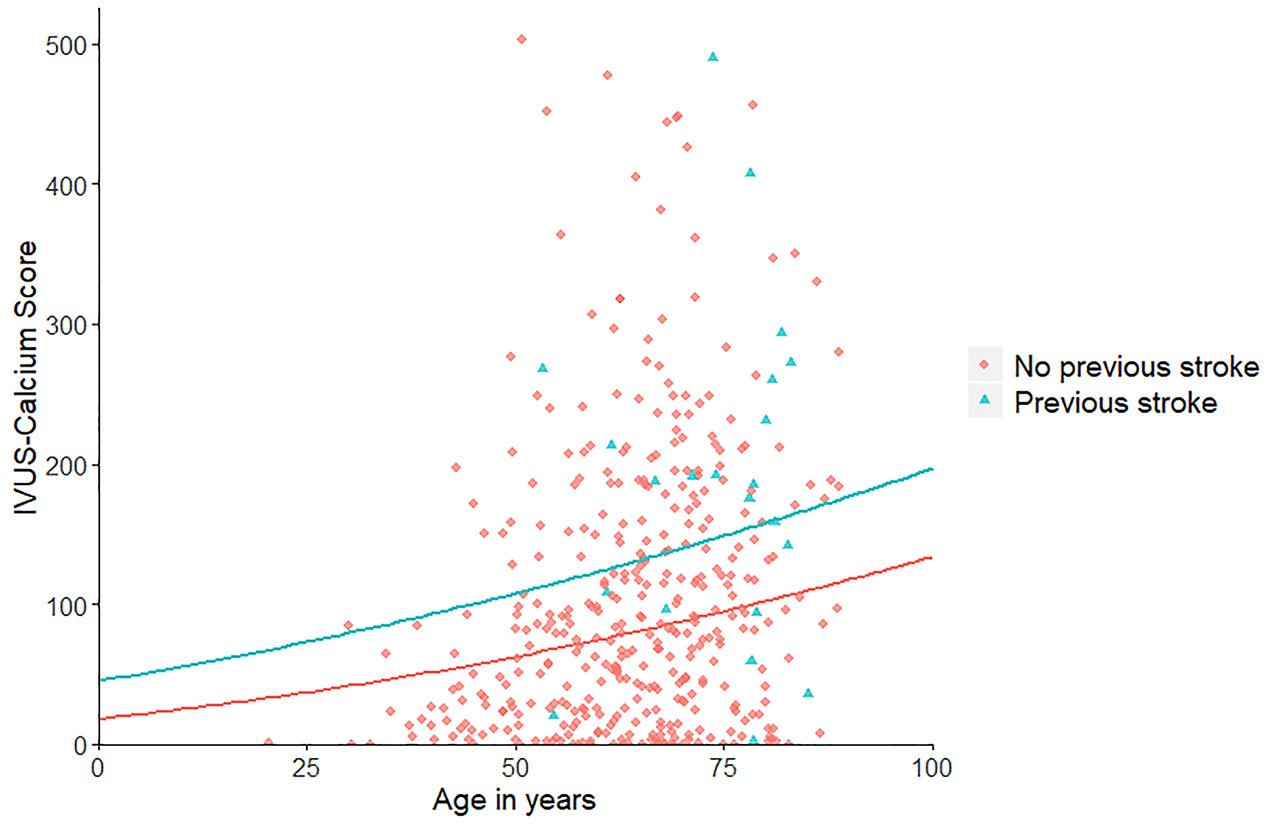

Supplement: Supplementary file 1 — Visual representation of relationship between ICS, age and a previous stroke on the linear scale. Regression coefficients were originally estimated for √ICS because of violation of the normality and homoscedasticity assumptions when using ICS, and then back-transformed by squaring the regression formula. Pink diamond shaped points represent the ICS and age of patients without a previous stroke. Blue triangle shaped points represent the ICS and age of patients with previous stroke. The blue and the pink line represent the regression line for the ICS of patients with varying age with or without a previous stroke, respectively. Abbreviations: ICS = IVUS-Calcium score. (PNG 3114 kb) [file 12265_2021_10103_Fig4_ESM.png]

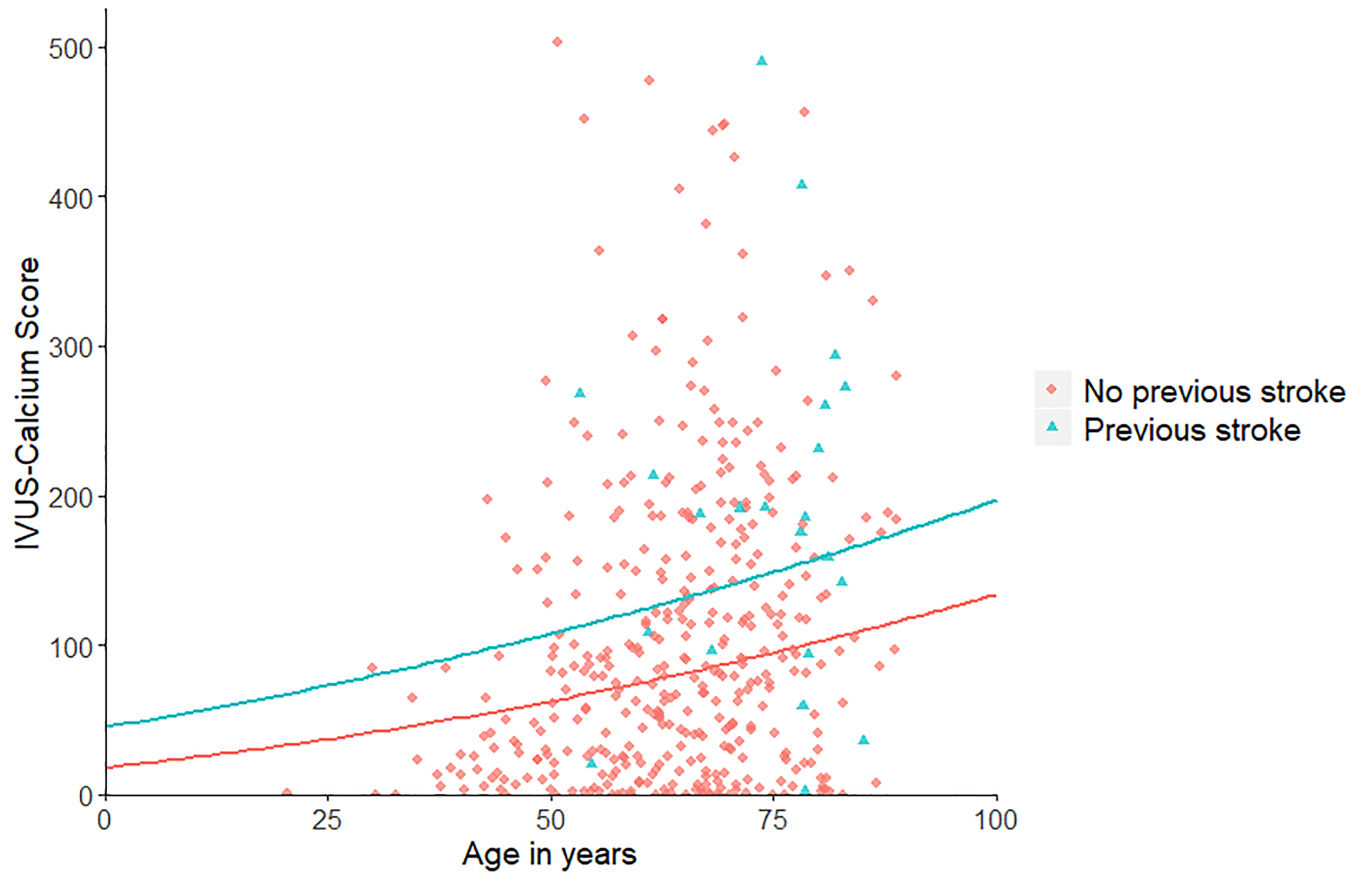

Supplement: Supplementary file 2 — High resolution image (TIF 12461 kb) [file 12265_2021_10103_MOESM1_ESM.tif]
