# Supplementary material for: The Prognostic Value of a Validated and Automated Intravascular Ultrasound-Derived Calcium Score
Source: J Cardiovasc Transl Res. 2021 Feb 23;14(5):992–1000. doi: 10.1007/s12265-021-10103-1 (PMC8575752; doi:10.1007/s12265-021-10103-1)
Supplement: Supplementary file 3 — (DOCX 19 kb) [file 12265_2021_10103_MOESM2_ESM.docx]

**Electronic Supplementary Material**

**The prognostic value of a validated and automated intravascular ultrasound-derived calcium score**

Journal of Translational Cardiovascular Research

Tara Neleman, BSc; Shengnan Liu, PhD; Maria N Tovar Forero, MD; Eline MJ Hartman, MD; Jurgen MR Ligthart, RT; Karen T Witberg, CCRN; Paul Cummins, MSc; Felix Zijlstra, MD, PhD; Nicolas M Van Mieghem, MD, PhD; Eric Boersma, PhD; Gijs van Soest, PhD; Joost Daemen, MD, PhD

**Corresponding author:**

Dr. J. Daemen

Department of Cardiology

Erasmus University Medical Center

Rotterdam, the Netherlands

j.daemen@erasmusmc.nl

**Supplementary Table 1:** Exclusion reasons and number of excluded pullbacks

| **Exclusion reason** | **Total screened IVUS pullbacks n = 1265** |
| --- | --- |
| Length < 40 mm | 355 |
| Stent struts present | 347 |
| Non-native coronary artery | 41 |
| Manual pullback | 39 |
| Shorter pullback of same patient, different coronary artery | 27 |
| Shorter pullback of same coronary artery | 24 |
| Poor imaging quality ^a^ | 9 |
| IVUS catheter in false lumen | 7 |
| Serial IVUS pullbacks in time ^b^ | 5 |
| Problems with computing ICS ^c^ | 3 |

^a^ Due to image artefacts, insufficient catheter preparation, and non-uniform rotational distortion etc.

^b^ When a patient underwent multiple IVUS pullbacks at different moments in time that met inclusion criteria, only the first pullback was included.

^c^ Dicom file issues.

Abbreviations: ICS = IVUS-calcium score, IVUS = intravascular ultrasound.

**Supplementary Table 2:** full multivariate cox regression models for the composite endpoint POCE and the secondary endpoints for both ICS ≥ 85 and ICS as a continuous score.

| **Cox regression** | **ICS ≥ 85** | | | **ICS_100 units_** | | |
| --- | --- | --- | --- | --- | --- | --- |
|  | **aHR** | **95% CI** | **P-value** | **aHR** | **95% CI** | **P-value** |
| **Model 1: multivariate model for POCE** This analysis was stratified for diabetes mellitus to solve non-proportional hazards | | | | | | |
| ICS | 1.51 | 1.05 – 2.17 | 0.026 | 1.21 | 1.04 – 1.41 | 0.014 |
| Gender | 0.92 | 0.63 – 1.34 | 0.66 | 0.91 | 0.63 – 1.32 | 0.62 |
| Age | 0.99 | 0.97 – 1.01 | 0.23 | 0.99 | 0.97 – 1.01 | 0.22 |
| Hypertension | 0.86 | 0.59 – 1.24 | 0.41 | 0.86 | 0.59 – 1.24 | 0.41 |
| Hypercholesterolemia | 0.97 | 0.68 – 1.37 | 0.86 | 1.01 | 0.72 – 1.43 | 0.94 |
| Smoking | 1.18 | 0.84 – 1.68 | 0.34 | 1.17 | 0.82 – 1.65 | 0.38 |
| Previous PCI | 0.91 | 0.57 – 1.44 | 0.69 | 0.90 | 0.56 – 1.43 | 0.65 |
| Previous MI | 0.93 | 0.60 – 1.45 | 0.76 | 0.93 | 0.60 – 1.45 | 0.74 |
| Previous CABG | 2.45 | 1.24 – 4.85 | 0.010 | 2.22 | 1.11 – 4.43 | 0.024 |
| Previous stroke | 1.17 | 0.60 – 2.30 | 0.65 | 1.20 | 0.61 – 2.35 | 0.60 |
| Previous PAD | 1.40 | 0.87 – 2.26 | 0.17 | 1.37 | 0.85 – 2.22 | 0.20 |
| eGFR | 0.99 | 0.98 – 1.00 | 0.002 | 0.99 | 0.98 – 0.99 | 0.001 |
| Presentation with ACS | 1.73 | 1.21 – 2.46 | 0.003 | 1.71 | 1.20 – 2.44 | 0.003 |
| IVUS vessel treated | 0.94 | 0.63 – 1.42 | 0.78 | 0.99 | 0.67 – 1.49 | 0.98 |
| **Model 2: multivariate model for any revascularization** | | | | | | |
| ICS | 1.31 | 0.82 – 2.10 | 0.26 | 1.23 | 1.00 – 1.51 | 0.055 |
| Gender | 0.90 | 0.54 – 1.50 | 0.69 | 0.92 | 0.55 – 1.53 | 0.75 |
| Age | 0.96 | 0.94 – 0.98 | < 0.001 | 0.96 | 0.94 – 0.98 | < 0.001 |
| Hypercholesterolemia | 1.39 | 0.86 – 2.26 | 0.18 | 1.45 | 0.90 – 2.33 | 0.13 |
| Previous PCI | 1.04 | 0.59 – 1.86 | 0.88 | 1.01 | 0.57 – 1.79 | 0.98 |
| Previous MI | 0.73 | 0.41 – 1.32 | 0.31 | 0.73 | 0.41 – 1.30 | 0.28 |
| Previous CABG | 4.07 | 1.87 – 8.84 | < 0.001 | 3.82 | 1.74 – 8.39 | < 0.001 |
| Previous PAD | 1.87 | 0.98 – 3.55 | 0.056 | 1.77 | 0.93 – 3.37 | 0.085 |
| **Model 3: multivariate model for PCI** | | | | | | |
| ICS | 1.25 | 0.77 – 2.05 | 0.37 | 1.20 | 0.96 – 1.49 | 0.11 |
| Gender | 0.81 | 0.48 – 1.39 | 0.44 | 0.83 | 0.49 – 1.42 | 0.50 |
| Age | 0.96 | 0.94 – 0.98 | < 0.001 | 0.96 | 0.94 – 0.98 | < 0.001 |
| Hypercholesterolemia | 1.45 | 0.87 – 2.43 | 0.15 | 1.50 | 0.91 – 2.49 | 0.11 |
| Smoking | 1.34 | 0.83 -2.19 | 0.23 | 1.33 | 0.82 – 2.16 | 0.25 |
| Previous PCI | 1.06 | 0.63 – 1.79 | 0.82 | 1.04 | 0.62 – 1.76 | 0.88 |
| Previous CABG | 5.15 | 2.35 – 11.27 | <0.001 | 4.85 | 2.20 – 10.69 | < 0.001 |
| **Model 4: univariate model for CABG** | | | | | | |
| ICS | 1.61 | 0.53 – 4.91 | 0.41 | 1.30 | 0.82 – 2.06 | 0.27 |
| **Model 5: multivariate model for target vessel recascularization** | | | | | | |
| ICS | 1.68 | 0.88 – 3.19 | 0.11 | 1.37 | 1.06 – 1.77 | 0.017 |
| Gender | 0.94 | 0.47 – 1.88 | 0.87 | 0.99 | 0.49 – 1.98 | 0.98 |
| Age | 0.96 | 0.93 – 0.98 | 0.002 | 0.96 | 0.93 – 0.98 | 0.001 |
| Previous CABG | 4.80 | 1.83 – 12.61 | 0.001 | 4.19 | 1.58 – 11.10 | 0.004 |
| **Model 6: multivariate model for myocardial infarction** | | | | | | |
| ICS | 1.45 | 0.69 – 3.02 | 0.33 | 1.28 | 0.95 – 1.73 | 0.10 |
| Previous stroke | 1.86 | 0.54 – 6.41 | 0.32 | 1.86 | 0.55 – 6.32 | 0.32 |
| Previous PAD | 1.87 | 0.75 – 4.72 | 0.18 | 1.86 | 0.68 – 4.39 | 0.25 |
| **Model 7: multivariate model for target vessel myocardial infarction** | | | | | | |
| ICS | 1.47 | 0.60 – 3.60 | 0.40 | 1.29 | 0.90 – 1.85 | 0.17 |
| Previous MI | 2.91 | 1.21 – 7.00 | 0.017 | 2.88 | 1.20 – 6.92 | 0.018 |
| **Model 8: univariate model for stroke** | | | | | | |
| ICS | 1.80 | 0.60 – 5.38 | 0.29 | 1.37 | 0.89 – 2.10 | 0.15 |
| **Model 9: multivariate model for all-cause mortality** | | | | | | |
| ICS | 1.55 | 0.91 – 2.64 | 0.10 | 1.16 | 0.94 – 1.43 | 0.17 |
| Age | 1.02 | 1.00 – 1.05 | 0.094 | 1.02 | 1.00 – 1.05 | 0.074 |
| Diabetes mellitus | 1.83 | 1.09 – 3.05 | 0.022 | 1.79 | 1.07 – 3.01 | 0.027 |
| Previous stroke | 1.07 | 0.45 – 2.56 | 0.88 | 1.11 | 0.46 – 2.66 | 0.81 |
| Previous PAD | 1.83 | 1.00 – 3.36 | 0.051 | 1.77 | 0.96 – 3.27 | 0.070 |
| eGFR | 0.98 | 0.96 – 0.99 | < 0.001 | 0.98 | 0.96 – 0.99 | < 0.001 |

Abbreviations: ACS = acute coronary syndrome, aHR = adjusted hazard ratio, CABG = coronary artery bypass graft, CI = confidence interval, eGFR = estimated glomerular filtration rate, ICS = IVUS-Calcium score, IVUS = intravascular ultrasound, MI = myocardial infarction, PAD = peripheral arterial disease, PCI = percutaneous coronary intervention, POCE = patient-oriented composite endpoint.

**Supplementary Fig. 1**

Visual representation of relationship between ICS, age and a previous stroke on the linear scale.
Regression coefficients were originally estimated for √ICS because of violation of the normality and homoscedasticity assumptions when using ICS, and then back-transformed by squaring the regression formula. Pink diamond shaped points represent the ICS and age of patients without a previous stroke. Blue triangle shaped points represent the ICS and age of patients with previous stroke. The blue and the pink line represent the regression line for the ICS of patients with varying age with or without a previous stroke, respectively

Abbreviations: ICS = IVUS-Calcium score
